# External validation and update of the J-ACCESS model in an Italian cohort of patients undergoing stress myocardial perfusion imaging

**DOI:** 10.1007/s12350-022-03173-4

**Published:** 2023-01-04

**Authors:** Mario Petretta, Rosario Megna, Roberta Assante, Emilia Zampella, Carmela Nappi, Valeria Gaudieri, Teresa Mannarino, Roberta Green, Valeria Cantoni, Adriana D’Antonio, Mariarosaria Panico, Wanda Acampa, Alberto Cuocolo

**Affiliations:** 1 IRCCS Synlab SDN, Via Gianturco 113, 80142 Naples, Italy; 2 Institute of Biostructure and Bioimaging, National Council of Research, Naples, Italy; 3 Department of Advanced Biomedical Sciences, University Federico II, Via Pansini 5, 80131 Naples, Italy

**Keywords:** CAD, SPECT, MPI, diagnostic and prognostic application

## Abstract

**Background:**

Cardiovascular risk models are based on traditional risk factors and investigations such as imaging tests. External validation is important to determine reproducibility and generalizability of a prediction model. We performed an external validation of t the Japanese Assessment of Cardiac Events and Survival Study by Quantitative Gated SPECT (J-ACCESS) model, developed from a cohort of patients undergoing stress myocardial perfusion imaging.

**Methods:**

We included 3623 patients with suspected or known coronary artery disease undergoing stress single-photon emission computer tomography (SPECT) myocardial perfusion imaging at our academic center between January 2001 and December 2019.

**Results:**

In our study population, the J-ACCESS model underestimated the risk of major adverse cardiac events (cardiac death, nonfatal myocardial infarction, and severe heart failure requiring hospitalization) within three-year follow-up. The recalibrations and updated of the model slightly improved the initial performance: C-statistics increased from 0.664 to 0.666 and Brier score decreased from 0.075 to 0.073. Hosmer–Lemeshow test indicated a logistic regression fit only for the calibration slope (*P* = .45) and updated model (*P* = .22). In the update model, the intercept, diabetes, and severity of myocardial perfusion defects categorized coefficients were comparable with J-ACCESS.

**Conclusion:**

The external validation of the J-ACCESS model as well as recalibration models have a limited value for predicting of three-year major adverse cardiac events in our patients. The performance in predicting risk of the updated model resulted superimposable to the calibration slope model.

**Supplementary Information:**

The online version contains supplementary material available at 10.1007/s12350-022-03173-4.

## Introduction

The evaluation of cardiovascular risk is based on traditional risk factors and data from clinical investigations such as imaging tests. This methodology is currently used to forecast the outcome of cardiovascular tests as well as risk of cardiac events.^1–9^ However, using these prediction models at different times or with different cohorts from which they derived, frequently they proved inadequate.^5,10,11^ The poor performance can be due to several factors. With regards to the time factor, in the last decades the prevention as well as the development of diagnostic and therapeutic techniques have reduced mortality and morbidity in cardiovascular patients.^12,13^ In several studies on temporal trend of single-photon emission computed tomography (SPECT) myocardial perfusion imaging, the total volume of performed studies declined, the number of traditional risk factors increased, and the prevalence of abnormal studies decreased.^14–19^ On the other hand, also a contemporary model can result inadequate when it is used with a different cohort. In particular, when a model results poor by external validation, a procedure of data adaptation consists in its recalibration.^20,21^ In this case, the risk evaluation is computed by one (additive) or more parameters (additive and multiplicative) that change the values of the intercept and covariate coefficients of the logistic regression, that represents the model. Instead, a model that became obsolete or remains poor can be updated using the same variables of the model but with new coefficients inferred from values observed in a new cohort. Therefore, new coefficients of the variables are obtained. In the present study we performed an external validation of the Japanese Assessment of Cardiac Events and Survival Study by Quantitative Gated SPECT (J-ACCESS) model^7,8^ to evaluate its ability for predicting cardiac events using data from our institution. To obtain a complete validation, we performed two recalibrations and the update of the model.

## Methods

### Patients

We included a total of 3623 patients undergoing stress and rest SPECT myocardial perfusion imaging at our academic center between January 2001 and December 2019, with available follow-up for major adverse cardiac event (MACE), defined as cardiac death, nonfatal myocardial infarction, and severe heart failure requiring hospitalization within 3 years of the imaging study. These patients were part of an ongoing prospective dedicated database.^22^

The criteria used for patient selection were the same reported in the J-ACCESS study.^7,8^ More in detail, the inclusion criteria were ≥ 20 years of age and stress and rest ECG-gated SPECT performed for suspected or known coronary artery disease (CAD). Patients with onset of myocardial infarction or unstable angina pectoris within 3 months, valvular heart disease, idiopathic cardiomyopathy, severe arrhythmia, heart failure with class III or higher New York Heart Association classification, and severe liver or renal disorders were excluded. In agreement with the J-ACCESS protocol, we also excluded patients submitted to coronary artery revascularization within 60 days of the SPECT study. At the time of testing, clinical teams collected pertinent demographic and clinical information, past cardiac history, and CAD risk factors based on patient report or available medical records. Patients were classified as having diabetes if they were receiving treatment with oral hypoglycemic drugs or insulin. The review committee of our institution approved this study (Ethics Committee, University Federico II, protocol number 110/17), and all patients gave informed consent.

### Myocardial perfusion imaging

Patients underwent stress-optional rest ^99m^Tc-sestamibi SPECT myocardial perfusion imaging by physical exercise or pharmacologic stress using dipyridamole, according to the recommendations of the European Association of Nuclear Medicine.^23^ In all patients, beta-blocking medications and calcium antagonists were withhold for 48 hours and long-acting nitrates for 12 hours before testing. For patient undergoing exercise test, symptom-limited treadmill standardized protocols were performed. For dipyridamole stress test, patients were instructed not to consume products containing caffeine for 24 hours before the test. Dipyridamole was infused at dose of 0.142 mg⋅kg^−1^ min^−1^ intravenous over 4 minutes. A dose of 100 mg of aminophylline was administered intravenously in the event of chest pain or other symptoms, or after significant ST depression. At peak exercise, or 4 minutes after completion of dipyridamole infusion, patients were intravenously injected with 99mTc-sestamibi (8 to 10 mCi for stress and 32 to 40 mCi for rest). Imaging was started 30 to 45 minutes after tracer injection using a dual-head rotating gamma camera (E.CAM, Siemens Medical Systems, Hoffman Estates, IL, USA) equipped with a low-energy, high resolution collimator and connected with a dedicated computer system. No attenuation or scatter correction was used.

An automated software program (e-soft, 2.5, QGS/QPS, Cedars-Sinai Medical Center, Los Angeles, CA) was used to calculate left ventricular (LV) volumes and ejection fraction and the scores incorporating both the extent and severity of perfusion defects using standardized segmentation of 20 myocardial regions.^24^ Perfusion defects were quantified adding the scores of the 20 segments and expressed as summed stress score, representing the total myocardium abnormal. Summed stress and summed rest scores were measured independently from the stress and rest scans and summed difference score was defined as their difference. According to the J-ACCESS model, the severity of myocardial perfusion defects was defined with four grades of category (0, I, II, and III) using summed stress score: normal (score 0–3) and mildly (4–8), moderately (9–13), or severely (≥ 14) abnormal.^7,8^

### Statistical analysis

Statistical analysis was performed using the R software, version 6.3.3 (The R Foundation for Statistical Software, Vienna, Austria). Continuous variables were expressed as mean ± standard deviation and categorical data as percentages. Differences between groups were analyzed by Student *t* test or *χ*^2^ test, as appropriate. Two-sided *P* values < .05 were considered statistically significant. Marginal probability was defined as the percentage of patients with major adverse cardiac event with respect to the entire study population. For the external validation, we used the coefficients obtained from the J-ACCESS study.^7–9^ In particular, logit and probability were computed by the following formulas:

$$ logit = - 4.8125 + 0.8858 \left( {diabetes:0,1} \right) + 0.0558 \left( {age} \right) + 0.1941 \left( {SSS:0 - 3} \right) - 0.0475 \left( {LV ejection fraction} \right) $$

$$ p\left( \% \right) = \frac{1}{{1 + e^{ - logit} }} \times 100 $$

**Figure 1:**
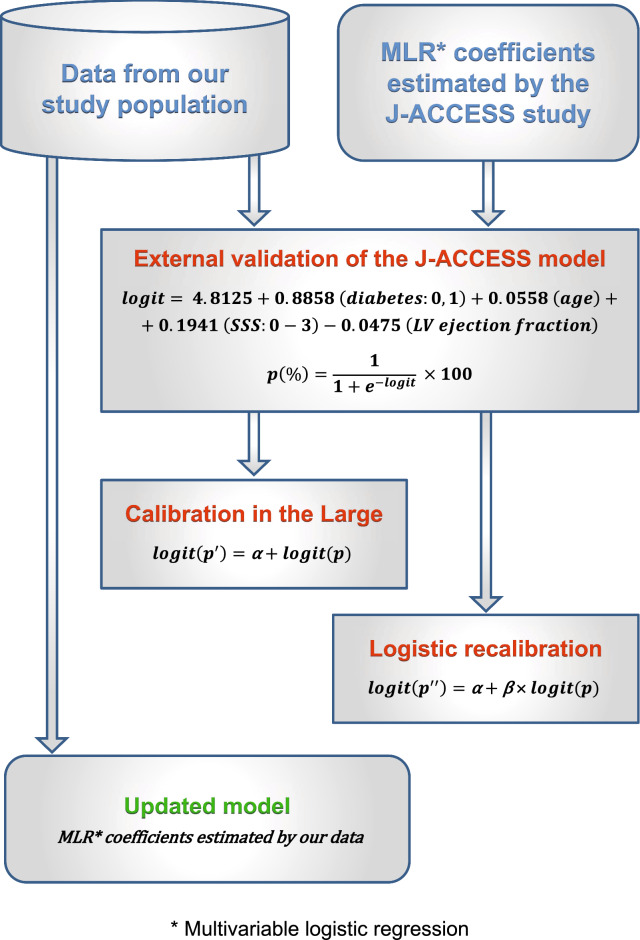
Flow chart of the study methods: external validation, recalibration procedures, and updated model

For the J-ACCESS external validation, we plotted the predicted probability across deciles versus the observed probability. These values were fitted by a linear regression, and the coefficient of determination (*R*^2^) was reported to evaluate the goodness-of-the-fit. On the same plot, we also reported the continuous values of the predicted versus observed variables, and the 95% confidence interval (CI). In order to obtain an exhaustive external validation, we recalibrated this model (logit (*p*)) with the calibration-in-the-large and the calibration slope (also called Logistic recalibration).^20,21^ In the first case, the statistic is given as the intercept term α from the recalibration model [logit (*p*′) = *α* + logit (*p*)] that changes baseline hazard. In the second case, *β* coefficient for all the variables and a new intercept are estimated from the recalibration model [logit (*p″*) = *α* + *β* × logit (*p*)]. We also realized an update of the J-ACCESS model by our data, using the same variables and computing new coefficients by a multivariable logistic regression with MACE as dependent variable. A flow chart of the study methods is sketched in Figure 1.

Statistic difference between initial and recalibration models was evaluated by residual deviance and likelihood ratio test (LRT) from *χ*^2^. We also computed the maximum (*d*_max_) and mean (*d*_mean_) difference in predicted vs loess-calibrated probabilities. For all the models, statistic concordance was calculated by the C-statistic, that represents the probability that patients with the outcome receive a higher predicted probability than those without. We also computed the Brier score that includes components of discrimination and calibration for models. This score improves decreasing from one to zero value. Finally, we evaluated the Hosmer–Lemeshow test, as a measure of goodness of fit, for predictive models of binary outcomes (logistic regressions), and sometimes used as a proxy for calibration.^25^ The 95% confidence interval (CI) for the continuous values of the predicted versus observed variables, *d*_max_, *d*_mean_, area under receiver operating characteristic curve, and Brier scores were computed by 1000 bootstrap resampling.

## Results

The percentage of our patients with MACE was 8.2%, significantly higher compared to the 4.3% found in the J-ACCESS study (*P* < .001). More in detail, we observed 140 (47%) cardiac deaths, 88 (30%) nonfatal myocardial infarctions, and 70 (23%) severe heart failures requiring hospitalization. The clinical characteristics and imaging findings of patients according to the occurrence of MACE are reported in Table 1. Patients with MACE had a higher prevalence of history of myocardial infarction or revascularization, diabetes and hypertension, and had a higher summed stress and rest scores and a lower LV ejection fraction than those without MACE.

**Table 1:** Clinical characteristics and imaging findings in patients with and without MACE

	No MACE (n = 3325)	MACE (n = 298)	*P* value
Age (years)	63 ± 11	67 ± 10	< .001
Male gender, n (%)	2184 (65)	211 (71)	.04
Typical chest pain, n (%)	630 (19)	44 (15)	.09
Body mass index, kg/m^2^	28.6 ± 13.8	28.1 ± 4.9	.15
History of myocardial infarction, n (%)	832 (25)	104 (35)	< .001
History of revascularization, n (%)	826 (25)	100 (34)	< .005
Diabetes, n (%)	1075 (32)	143 (48)	< .001
Hypertension, n (%)	2513 (76)	246 (83)	< .01
Hyperlipidemia, n (%)	1931 (58)	179 (60)	.54
Family history of CAD, n (%)	1316 (40)	128 (43)	.28
Currently smoking, n (%)	1015 (31)	103 (35)	.17
Ischemia during exercise ECG, n (%)	352 (11)	26 (9)	.36
Summed stress score	7.57 ± 8.97	10.5 ± 10.2	< .001
Summed rest score	4.71 ± 8.18	7.25 ± 10.0	< .001
Summed difference score	2.86 ± 4.19	3.25 ± 4.32	.14
LV end-diastolic volume (mL)	95 ± 47	117 ± 66	< .001
LV end-systolic volume (mL)	50 ± 39	69 ± 54	< .001
LV ejection fraction (%)	55 ± 12	50 ± 14	< .001

In our study population, myocardial perfusion was normal in 2177 (60%) and abnormal in 1446 (40%) patients. In these latter patients, 704 (19%) had mildly abnormal, 325 (9%) moderately abnormal, and 417 (12%) severely abnormal myocardial perfusion defects.

The statistics associated with the J-ACCESS, recalibration, and updated models are summarized in Table 2. For the calibration-in-the-large model *α* was 0.593, whereas for the calibration slope *α* was − 0.615 and *β* 0.564. The recalibrations improved the performance of the initial model. In fact, the residual deviance and *d*_mean_ decreased (from 2090 to 1962 and from 0.034 to 0.005, respectively). Also, the C-statistics and Brier score improved slightly (from 0.664 to 0.666 and from 0.075 to 0.073, respectively), indicating a little better discrimination and calibration of the models. Confirming this improvement, the models were all different compared to the initial one by the LRT test (*P* < .001). The Hosmer–Lemeshow test (calculated on deciles) indicated a logistic regression fit for the calibration slope (*P* = .45) and updated model (*P* = .22). High similarity between these two models we also found by LRT (*P* = .58). Lastly, the fits obtained by deciles between predicted and calculated probabilities resulted significant (*P* < .001) and highly correlates (*R*^2^ ≥ 0.90), highlighting a linear relationship.

**Table 2:** Statistics related to the J-ACCESS, recalibration, and updated models

	J-ACCESS model	Calibration in the large	Logistic recalibration	Revised model*
Intercept	0	0.593	− 0.615	–
Slope	1	1	0.564	–
Degrees of freedom	3623	3622	3621	3618
Residual deviance	2090	2015	1962	1960
*d*_max_	0.226 (0.055, 0.394)	0.354 (0.193, 0.488)	0.039 (0.012, 0.147)	0.033 (0.010, 0.139)
*d*_mean_	0.034 (0.026, 0.043)	0.024 (0.016, 0.032)	0.005 (0.002, 0.012)	0.003 (0.002, 0.010)
Likelihood ratio test *χ*^2^	–	*P* < .001	*P* < .001	*P* < .001
Hosmer–Lemeshow test *χ*^2^	214.9; *P* < .001	69.8; *P* < .001	7.8; *P* = .45	10.6; *P* = .22
Fit on deciles *R*^2^	0.938; *P* < .001	0.946; *P* < .001	0.941; *P* < .001	0.900; *P* < .001
C-statistic	0.664 (0.632, 0.696)	0.664 (0.632, 0.696)	0.664 (0.632, 0.698)	0.666 (0.638, 0.700)
Brier score	0.075 (0.066, 0.083)	0.075 (0.067, 0.083)	0.073 (0.066, 0.081)	0.073 (0.066, 0.08)

95% Confidence interval obtained by 1000 resampling bootstrap are reported in parentheses

*Intercept and coefficients of the revised model are reported in Table 3

**Figure 2:**
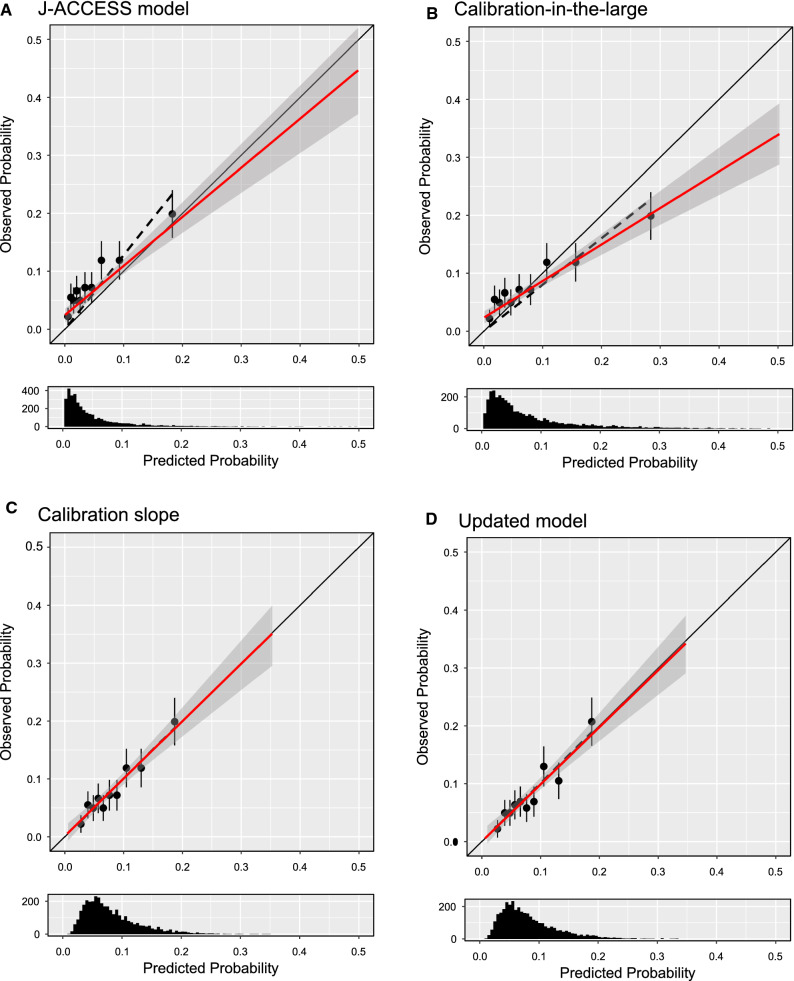
Predicted vs. observed probability for the J-ACCESS (panel **A**), calibration-in-the-large (panel **B**), calibration slope (panel **C**), and updated (panel **D**) models. The full circles represent probability expressed in deciles, whereas the error bars highlight the standard deviations. The black dashed line represents the linear regression calculated by deciles. The red line is related to the continuous values of the variables, with the 95% CI highlighted by the shaded zone. The diagonal represents the best agreement line between predicted and observed probabilities. The histogram in the graph below reports the predicted probability distribution

Figure 2 shows the predicted vs. observed probability for the J-ACCESS model (panel A), calibration-in-the-large (panel B), calibration slope (panel C), and updated model (panel D). These variables are presented both across deciles and as continuous. In general, the J-ACCESS model underestimates the observed data. For the calibration-in-the-large model is evident a shift toward the diagonal, with an underestimation for low values of the observed probability and an overestimation for those high. This effect was due to the intercept (*α*) introduced in the model. The logistic recalibration shows a better fit of the data than the initial model, mainly due to the *β* coefficient that causes a compression of probabilities. The updated model was no more performing than the recalibrated logistic model, highlighting a limited value of the J-ACCESS variables.

The multivariable logistic regression findings related to the updated model are reported in Table 3. All the independent variables resulted significant (*P* < .01). With respect to age, the risk of MACE increased by 4% for each year. Patients with diabetes were found to have a 73% higher risk than those without. For summed stress score the risk was 16% higher for each gap between two categories. As expected, the increase in LV ejection fraction was protective (2% for each percent of increment). Within 3 standard errors, intercept, diabetes, and summed stress score categorized coefficients of the updated model were comparable with J-ACCESS ones.

**Table 3:** Multivariable logistic regression for MACE

	Estimate	Standard error	*P* value	Odds ratio (95% CI)
Intercept	− 3.8988	0.4986	< .001	–
Diabetes	0.5482	0.1238	< .001	1.73 (1.36, 2.20)
Age	0.0348	0.0065	< .001	1.04 (1.02, 1.05)
Summed stress score*	0.1452	0.0576	< .01	1.16 (1.03, 1.29)
LV ejection fraction	− 0.0211	0.0050	< .001	0.98 (0.97, 0.99)

*Summed stress score was categorized as normal (score 0–3), mildly (score 4–8), moderately (score 9–13), or severely (score ≥ 14) abnormal

To assess the time effects on the patient characteristics, we split our study population into group 1 (from 2001 to 2010, n = 1788) and group 2 (from 2011 to 2019, n = 1835). For group 1, more contemporary to the J-ACCESS model, we found a slightly better adaptation of data to the model (see Tables S1, S3 in the supplementary materials). There was a significant difference between the two groups only comparing the Brier score (0.052 vs 0.096). This difference was due to the lower number of events in patients of group 1 compared to those of group 2 (5.5% vs 10.8%). Investigating this difference, we found a higher number of patients with abnormal summed stress score in group 1 compared to group 2 (51% vs 70%). Regarding the updated models, group 1 and group 2 were superimposable, highlighting similar characteristics with respect to the J-ACCESS variables. In fact, the *P* resulted different in absolute value, but with same statistical significance (see Table S2 and Table S4 in the supplementary materials).

For the entire study population, we also evaluated a more complete updated model considering as covariates in the multivariable logistic regression the J-ACCESS variables and traditional risk factors (angina, dyspnea, gender, hyperlipidemia, hypertension, and smoking). We did not find significant differences between this model and the updated model with only the J-ACCESS variables (see Table S5 in the Supplementary materials).

## Discussion

The present study was designed to obtain an external validation and an update of the J-ACCESS model, for predicting three-year major cardiac events in patients with and without history of CAD. Comparing our and J-ACCESS cohorts it is important to highlight that the variables between the group of patients with and without events showed significant different for body mass index, history of coronary artery revascularization, and hypertension. It should also be noted that the characteristics of J-ACCESS patients were obtained for hard events, i.e., without considering patients with heart failure requiring hospitalization. However, from the point of view of patients’ characteristics our and J-ACCESS cohorts were quite similar. Instead, a difference between the two cohorts was due to the percentage of patients with outcome that in J-ACCESS study was significantly lower than in our study. This finding is in line with a lower cardiovascular risk in Japan than in Italy.^26^

Using the J-ACCESS model in our study population, we observed an underestimation of the predicted risk compared to the observed risk. The effect is evident from the graph of predicted versus observed probabilities, where the full circles representing deciles are over the diagonal (i.e., equal probability line). We can likely explain the non-optimal fit of the J-ACCESS model to our data on the bases of the different marginal probability between the two cohorts.

To obtain a more accurate external validation, we considered the calibration-in-the-large and the logistic recalibration models. The term of intercept computed by the calibration-in-the-large model did not significantly improve the external validation findings. Instead, using the logistic recalibration model, we observed a better agreement between predicted versus observed probability. As a matter of fact, in comparison with the two previous models we obtained the lower *d*_mean_, the Hosmer–Lemeshow test indicated a good adaptation of the logistic regression fit to data, and the Brier score slightly increased. The improvement is explainable by the change in profile of the predicted probability distribution due to α and β terms of the recalibrated model. In general, nevertheless the models resulted different to the LRT, they did not show a significant improvement to the C-statistic and Brier score. Therefore, to verify the predictivity of the independent variables we also evaluated an update of the J-ACCESS model. As expected, we observed a good agreement between predicted and observed probabilities. However, we did not find significant improvements than to the calibration slope model by the statistics. In fact, the two model are superimposable with respect to all performed tests. This result brings to lite two aspects. On the one hand, we can infer that the features used for the model have a limited value for the prediction of risk. On the other hand, it indicates that the recalibration model used is an effective method because gives findings very similar to the model obtained with an internal cohort.

The time effects on the study population characteristics were marginal, explainable by higher number of patients with major burden of disease in the more recent years. This situation was probably due to the greater appropriateness of clinical tests adopted in the last years.

Adding traditional risk factors to the updated model we did not find other significant variables. This result highlights that age and diabetes are predominant traditional risk factors. In fact, they, together with summed stress score and LV ejection fraction, adjust the other covariates making those not significant at the multivariable logistic regression nevertheless some of them resulted significant to the *χ*^2^ test (e.g., gender and hypertension).

With regards to the external validation related to major cardiac events, in another study we evaluated the performance of the CRAX2MACE model.^11^ Unlike what has happened in the present study, the risk evaluation was within two-year and only included subjects with suspect CAD. Moreover, the study was conducted in North America. The external validation of the CRAX2MACE model with our data resulted in overestimating the risk and the recalibration models did not give better results than the initial one.

The findings obtained through this study and previous investigations confirm that any prediction model performs best in the population it was derived from. Therefore, an optimal model for predicting of major cardiac events adaptable to generalized data does not seem currently available. Likely, a multicenter model obtained by a large amount of heterogeneous data, also supported from machine learning techniques, could carried out a more efficacy model. In particular, this technique of artificial intelligence is finding many applications from clinical imaging to pretest as a support to diagnosis in different medical fields, also with specific recommendations in cardiology.^27–31^

Another multicenter study, based on traditional risk factors, was conducted to estimate 10-year risk of cardiovascular disease in Europe (SCORE2).^32^ The authors derived risk prediction models using individual-participant data from 45 cohorts (677,684 individuals, 30,121 cardiovascular disease events), and defined four risk regions in Europe according to country-specific cardiovascular disease mortality, recalibrating models to each region using expected incidences and risk factor distributions. In particular, region-specific incidence was obtained by data over ten millions of persons, and for the external validation were used data from 25 additional cohorts (over one million of individuals, and 43,492 cardiovascular events). After applying the derived risk prediction models to external validation cohorts, C-statistic ranged from 0.67 (0.65–0.68) to 0.81 (0.76–0.86). These C-statistic values are in line with, or slightly better than, the cited studies based on traditional risk factors.

To improve the performance of cardiologic forecasting models, it may be necessary to find novel variables. An example of multicenter study obtained using non-traditional risk factors and finalized to fatal cardiovascular disease risk prediction was reported by Tillmann et al.^33^ Patients from various Eastern European states took part to the derivation cohort (~ 14,600), and patients from Estonia formed the validation cohort (~ 4,600). In this study three models were evaluated, also using variables such as education and depression. The authors found the area under receiver operating characteristic curve in the range 78–87%, with better values in the validation cohort.

In general, the research of new features is desirable also in other areas of cardiology. For example, in subjects with zero-calcium score who underwent coronary artery computer tomography, the predicted models were unable to identify individuals with a very low probability of an abnormal stress myocardial perfusion imaging.^34^ Efficiency of models in cardiology remains a field still to be improved, and the use of strategies as multicenter data, novel features, and machine learning techniques seems to be an interesting way to go.

To the best of our knowledge, the errors on the J-ACCESS intercept and coefficients are not available.^7,8^ Therefore, we compared our updated model with the J-ACCESS model considering our estimates of parameters and standard errors vs. the J-ACCESS parameters alone, without their errors. Patients’ characteristics in our study were computed with respect to major adverse cardiac events, while in J-ACCESS^7^ were computed for patients with “hard cardiac events”, that is only using cardiac death and nonfatal myocardial infarction. Our choice was due to the fact that the J-ACCESS parameters used in this study were given in several articles where were considered patients with major adverse cardiac events.^7–9,35–37^

## New knowledge gained

This study confirms that clinical prediction models perform best in the population they were derived from. In addition, it supports the use of recalibration of predictive models to improve the model performance on external cohorts. Based on changes in diagnostic and therapeutic approaches for many diseases, predictive models may lose efficacy on time. Therefore, external validation and updating of the models in different cohorts is worthwhile to address the reproducibility and generalizability of any clinical prediction model.

## Conclusion

External validation of a predictive model is necessary to determine reproducibility and generalizability to new cohorts. This method is gaining increasing importance for considering a model clinically acceptable. The results of this study indicate that the J-ACCESS model have a limited value for predicting of three-year major cardiac events in our study population. The performance in predicting risk of the updated model resulted superimposable to the calibration slope model.

## Supplementary Information

Below is the link to the electronic supplementary material.

Supplementary file1 (DOC 105 kb)

Supplementary file2 (PPTX 225 kb)

Supplementary file3 (PPTX 332 kb)

Supplementary file4 (MP3 4966 kb)

## Abbreviations

SPECT: Single-photon emission computed tomography
J-ACCESS: Japanese Assessment of Cardiac Events and Survival Study by Quantitative Gated SPECT
MACE: Major adverse cardiac event
CAD: Coronary artery disease
LV: Left ventricular
*d*_max_: Maximum difference in predicted vs. loess-calibrated probabilities
*d*_mean_: Mean difference in predicted vs. loess-calibrated probabilities
CI: Confidence interval
