# Supplementary material for: External validation and update of the J-ACCESS model in an Italian cohort of patients undergoing stress myocardial perfusion imaging
Source: J Nucl Cardiol. 2023 Jan 4;30(4):1443–53. doi: 10.1007/s12350-022-03173-4 (PMC10371932; doi:10.1007/s12350-022-03173-4)
Supplement: Supplementary file 1 — Supplementary file1 (DOC 105 kb) [file 12350_2022_3173_MOESM1_ESM.doc]

**Table S1.** Statistics related to the J-ACCESS, recalibration and updated models for 1788 patients who underwent SPECT from 2001 to 2010

^*^Intercept and coefficients of the revised model are reported in Table S4

|  | **J-ACCESS model** | **Calibration in the large** | **Logistic recalibration** | **Revised model*** |
| --- | --- | --- | --- | --- |
| Intercept | 0 | 0.217 | -1.03 | − |
| Slope | 1 | 1 | 0.554 | − |
| Degrees of freedom | 1788 | 1787 | 1786 | 1783 |
| Residual deviance | 756 | 752 | 732 | 731 |
| d_max_ | 0.395 (0.173, 0.507) | 0.436 (0.209, 0.555) | 0.105 (0.023, 0.237) | 0.09 (0.015, 0.202) |
| d_mean_ | 0.017 (0.011, 0.027) | 0.018 (0.01, 0.028) | 0.003 (0.003, 0.011) | 0.003 (0.003, 0.012) |
| Likelihood ratio test χ^2^ | − | *P* < .05 | *P* < .001 | *P* < .001 |
| Hosmer-Lemeshow test χ^2^ | 41.2; *P* < .001 | 30.2; *P* < .001 | 8.35; *P* = 0.40 | 9.04; *P* = 0.34 |
| Fit on deciles R^2^ | 0.818; *P* < .001 | 0.819; *P* < .001 | 0.833; *P* < .001 | 0.841; *P* < .001 |
| C-statistic | 0.666 (0.617, 0.72) | 0.665 (0.609, 0.716) | 0.665 (0.611, 0.717) | 0.670 (0.626, 0.725) |
| Brier score | 0.052 (0.043, 0.061) | 0.053 (0.044, 0.063) | 0.051 (0.042, 0.061) | 0.051 (0.042, 0.061) |

95% Confidence interval obtained by 1000 resampling bootstrap are reported in parentheses

**Table S2.** Multivariable logistic regression for MACE in patients who underwent SPECT from 2001 to 2010

|  | **Estimate** | **Standard error** | ***P*-value** | **Odds ratio (95% CI)** |
| --- | --- | --- | --- | --- |
| Intercept | -4.1336 | 0.8463 | <.001 | − |
| Diabetes | 0.4227 | 0.2125 | <.05 | 1.53 (1.00, 2.31) |
| Age | 0.0332 | 0.0111 | <.005 | 1.03 (1.01, 1.06) |
| Summed stress score^*^ | 0.1777 | 0.0985 | 0.07 | 1.19 (0.98, 1.44) |
| LV ejection fraction | -0.0213 | 0.0087 | <.05 | 0.98 (0.96, 1.00) |

^*^Summed stress score was categorized as normal (score 0-3), mildly (score 4-8), moderately (score 9-13), or severely (score  ≥14) abnormal

**Table S3.** Statistics related to the J-ACCESS, recalibration and updated models for 1835 patients who underwent SPECT from 2011 to 2019

^*^Intercept and coefficients of the revised model are reported in Table S4

|  | **J-ACCESS model** | **Calibration in the large** | **Logistic recalibration** | **Revised model*** |
| --- | --- | --- | --- | --- |
| Intercept | 0 | 0.851 | -0.411 | − |
| Slope | 1 | 1 | 0.542 | − |
| Degrees of freedom | 1835 | 1834 | 1833 | 1830 |
| Residual deviance | 1335 | 1239 | 1203 | 1202 |
| d_max_ | 0.08 (0.066, 0.31) | 0.282 (0.104, 0.454) | 0.022 (0.014, 0.175) | 0.022 (0.014, 0.172) |
| d_mean_ | 0.055 (0.043, 0.07) | 0.031 (0.02, 0.044) | 0.008 (0.003, 0.019) | 0.008 (0.003, 0.019) |
| Likelihood ratio test χ^2^ | − | *P* < .001 | *P* < .001 | *P* < .001 |
| Hosmer-Lemeshow test χ^2^ | 238; *P* < .001 | 49.4; *P* < .001 | 4.95; *P* = 0.76 | 6.67; *P* = 0.57 |
| Fit on deciles R^2^ | 0.948; *P* < .001 | 0.958; *P* < .001 | 0.943; *P* < .001 | 0.905; *P* < .001 |
| C-statistic | 0.655 (0.611, 0.698) | 0.654 (0.611, 0.694) | 0.654 (0.61, 0.696) | 0.657 (0.62, 0.703) |
| Brier score | 0.096 (0.084, 0.109) | 0.095 (0.084, 0.107) | 0.093 (0.082, 0.104) | 0.093 (0.083, 0.103) |

95% Confidence interval obtained by 1000 resampling bootstrap are reported in parentheses

**Table S4.** Multivariable logistic regression for MACE in patients who underwent SPECT from 2011 to 2019

|  | **Estimate** | **Standard error** | ***P*-value** | **Odds ratio (95% CI)** |
| --- | --- | --- | --- | --- |
| Intercept | -3.3315 | 0.6211 | <.001 | − |
| Diabetes | 0.6269 | 0.1536 | <.001 | 1.87 (1.38, 2.53) |
| Age | 0.0313 | 0.0081 | <.001 | 1.03 (1.02, 1.05) |
| Summed stress score^*^ | 0.0902 | 0.0723 | 0.21 | 1.09 (0.95, 1.26) |
| LV ejection fraction | -0.0220 | 0.0062 | <.001 | 0.98 (0.97, 0.99) |

^*^Summed stress score was categorized as normal (score 0-3), mildly (score 4-8), moderately (score 9-13), or severely (score  ≥14) abnormal

**Table S5.** Multivariable logistic regression for MACE considering as covariates the J-ACCESS variables and traditional risk factors in the overall study population

|  | **Estimate** | **Standard error** | ***P*-value** | **Odds ratio (95% CI)** |
| --- | --- | --- | --- | --- |
| Intercept | -4.1644 | 0.5328 | <.001 | − |
| Diabetes | 0.5577 | 0.1254 | <.001 | 1.75 (1.37, 2.23) |
| Age | 0.0344 | 0.0067 | <.001 | 1.04 (1.02, 1.05) |
| Summed stress score^*^ | 0.1380 | 0.0588 | <.05 | 1.15 (1.02, 1.29) |
| LV ejection fraction | -0.0209 | 0.0051 | <.001 | 0.98 (0.97, 0.99) |
| Angina | -0.0482 | 0.1381 | 0.73 | 0.95 (0.72, 1.25) |
| Dyspnea | 0.2355 | 0.1444 | 0.10 | 1.27 (0.95, 1.67) |
| Gender | 0.0642 | 0.1412 | 0.65 | 1.07 (0.81, 1.41) |
| Hyperlipidemia | -0.1036 | 0.1292 | 0.42 | 0.90 (0.70, 1.16) |
| Hypertension | 0.2290 | 0.1656 | 0.17 | 1.26 (0.92, 1.75) |
| Smoking | 0.2382 | 0.1341 | 0.08 | 1.27 (0.97, 1.65) |

^*^Summed stress score was categorized as normal (score 0-3), mildly (score 4-8), moderately (score 9-13), or severely (score  ≥14) abnormal
