# Supplementary material for: External validation and update of the J-ACCESS model in an Italian cohort of patients undergoing stress myocardial perfusion imaging
Source: J Nucl Cardiol. 2023 Jan 4;30(4):1443–53. doi: 10.1007/s12350-022-03173-4 (PMC10371932; doi:10.1007/s12350-022-03173-4)
Supplement: Supplementary file 2 — Supplementary file2 (PPTX 225 kb) [file 12350_2022_3173_MOESM2_ESM.pptx]

## Slide 1
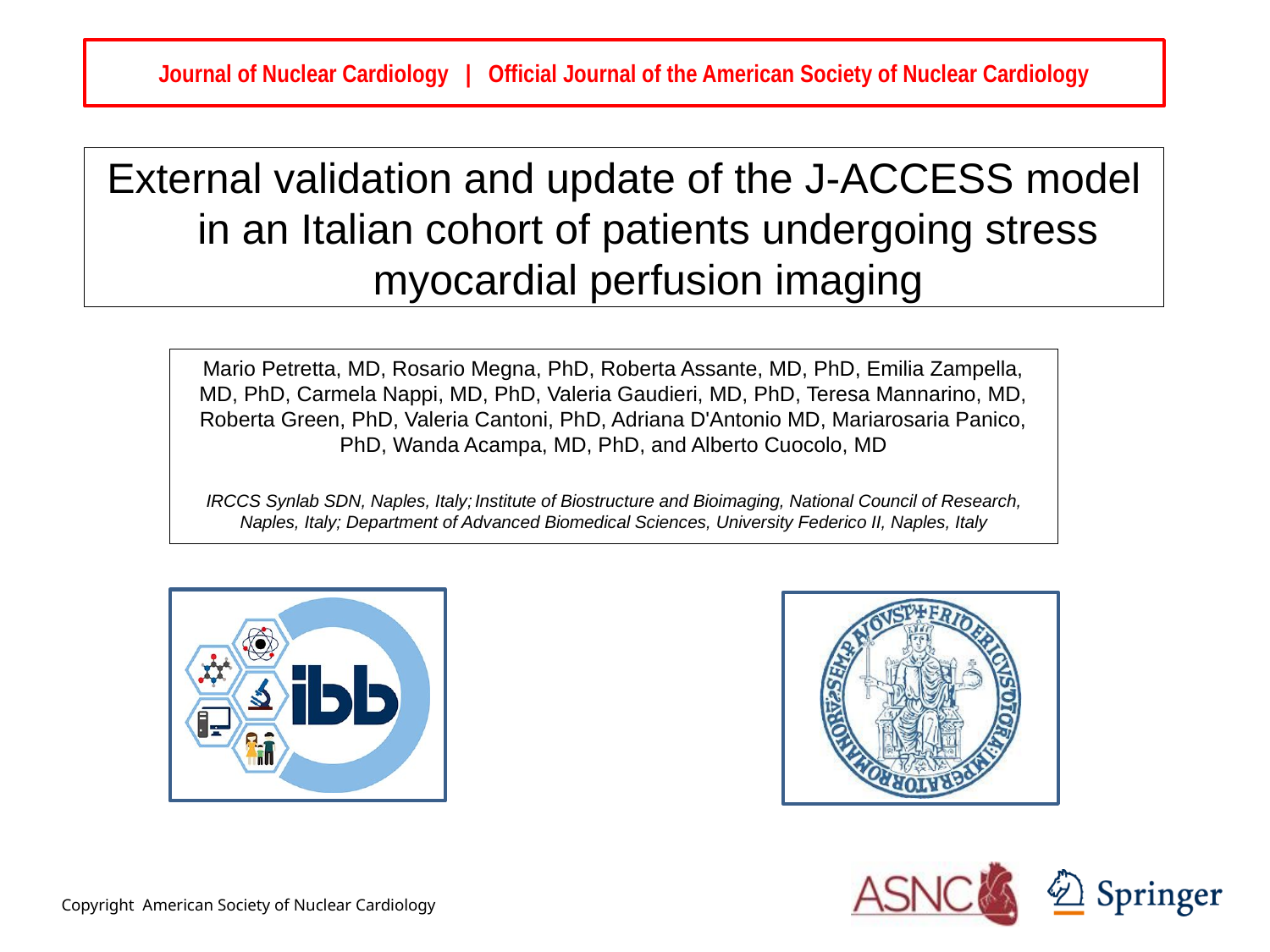

Journal of Nuclear Cardiology | Official Journal of the American Society of Nuclear Cardiology
# External validation and update of the J-ACCESS model in an Italian cohort of patients undergoing stress myocardial perfusion imaging
Mario Petretta, MD, Rosario Megna, PhD, Roberta Assante, MD, PhD, Emilia Zampella, MD, PhD, Carmela Nappi, MD, PhD, Valeria Gaudieri, MD, PhD, Teresa Mannarino, MD, Roberta Green, PhD, Valeria Cantoni, PhD, Adriana D'Antonio MD, Mariarosaria Panico, PhD, Wanda Acampa, MD, PhD, and Alberto Cuocolo, MD
IRCCS Synlab SDN, Naples, Italy; Institute of Biostructure and Bioimaging, National Council of Research, Naples, Italy; Department of Advanced Biomedical Sciences, University Federico II, Naples, Italy
Head shot of author
required
Copyright American Society of Nuclear Cardiology

## Slide 2
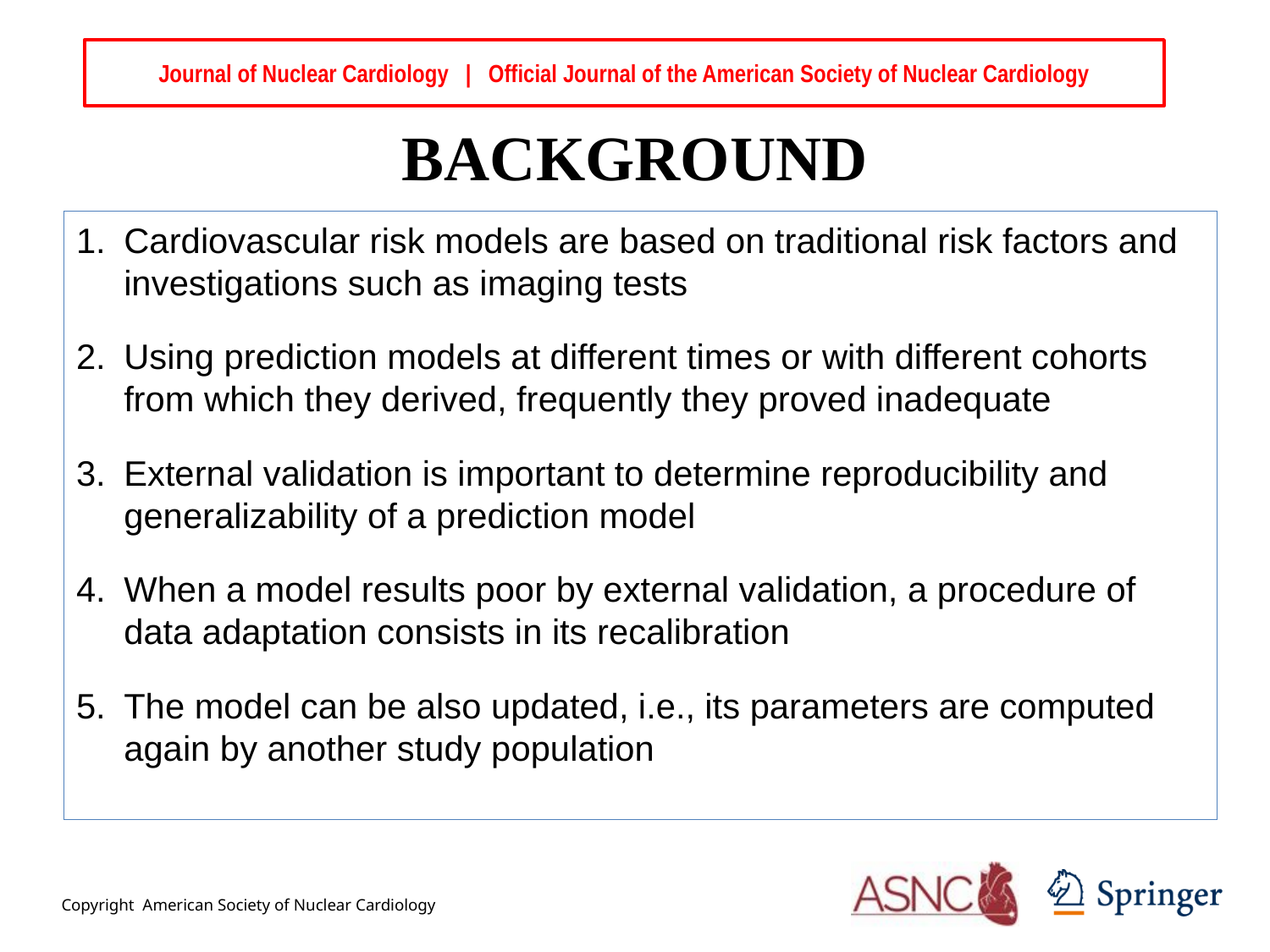

Journal of Nuclear Cardiology | Official Journal of the American Society of Nuclear Cardiology
# BACKGROUND
Cardiovascular risk models are based on traditional risk factors and investigations such as imaging tests
Using prediction models at different times or with different cohorts from which they derived, frequently they proved inadequate
External validation is important to determine reproducibility and generalizability of a prediction model
When a model results poor by external validation, a procedure of data adaptation consists in its recalibration
The model can be also updated, i.e., its parameters are computed again by another study population
Copyright American Society of Nuclear Cardiology

## Slide 3
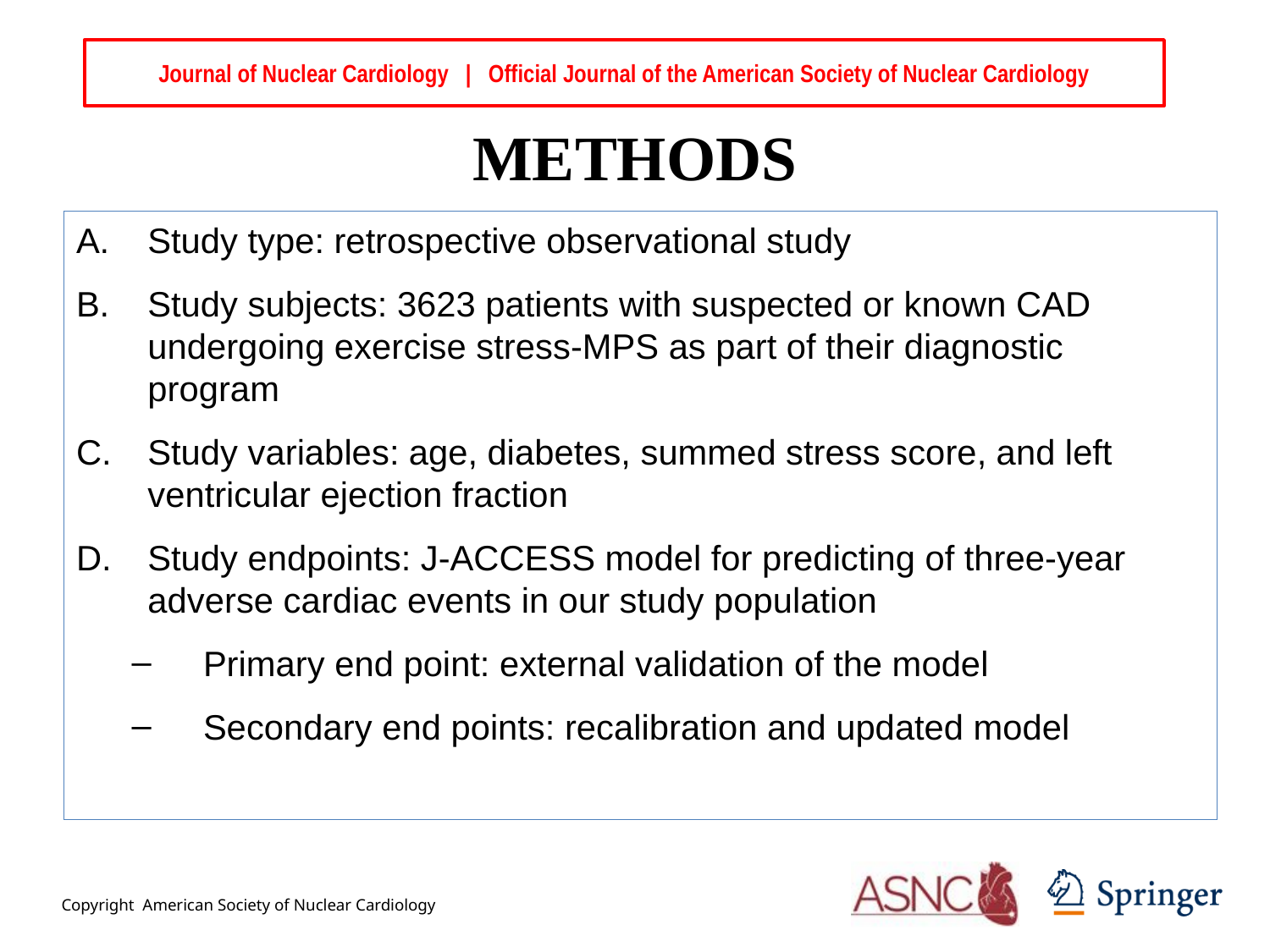

Journal of Nuclear Cardiology | Official Journal of the American Society of Nuclear Cardiology
# METHODS
Study type: retrospective observational study
Study subjects: 3623 patients with suspected or known CAD undergoing exercise stress-MPS as part of their diagnostic program
Study variables: age, diabetes, summed stress score, and left ventricular ejection fraction
Study endpoints: J-ACCESS model for predicting of three-year adverse cardiac events in our study population
Primary end point: external validation of the model
Secondary end points: recalibration and updated model
Copyright American Society of Nuclear Cardiology

## Slide 4
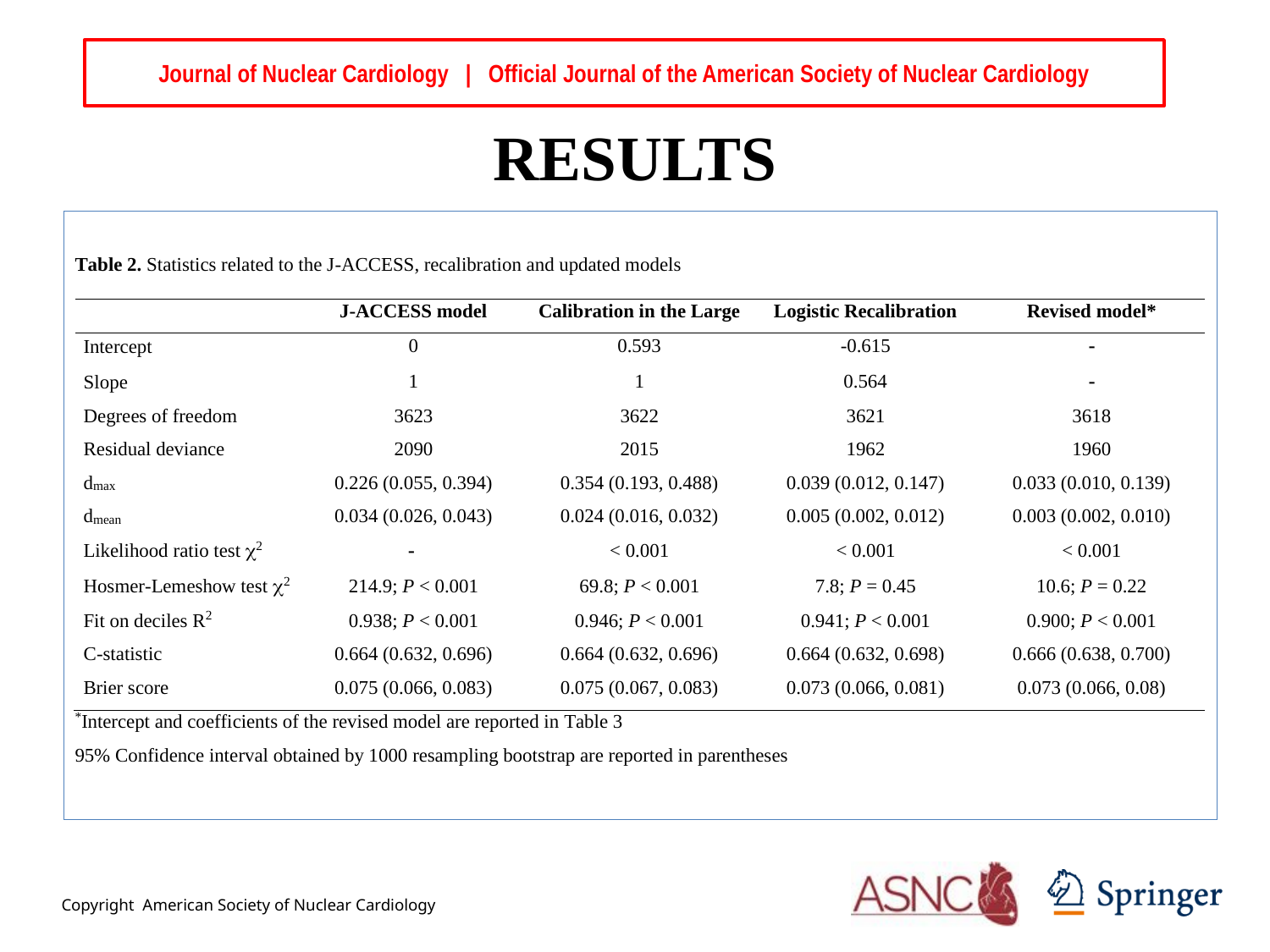

Journal of Nuclear Cardiology | Official Journal of the American Society of Nuclear Cardiology
# RESULTS
Copyright American Society of Nuclear Cardiology

## Slide 5
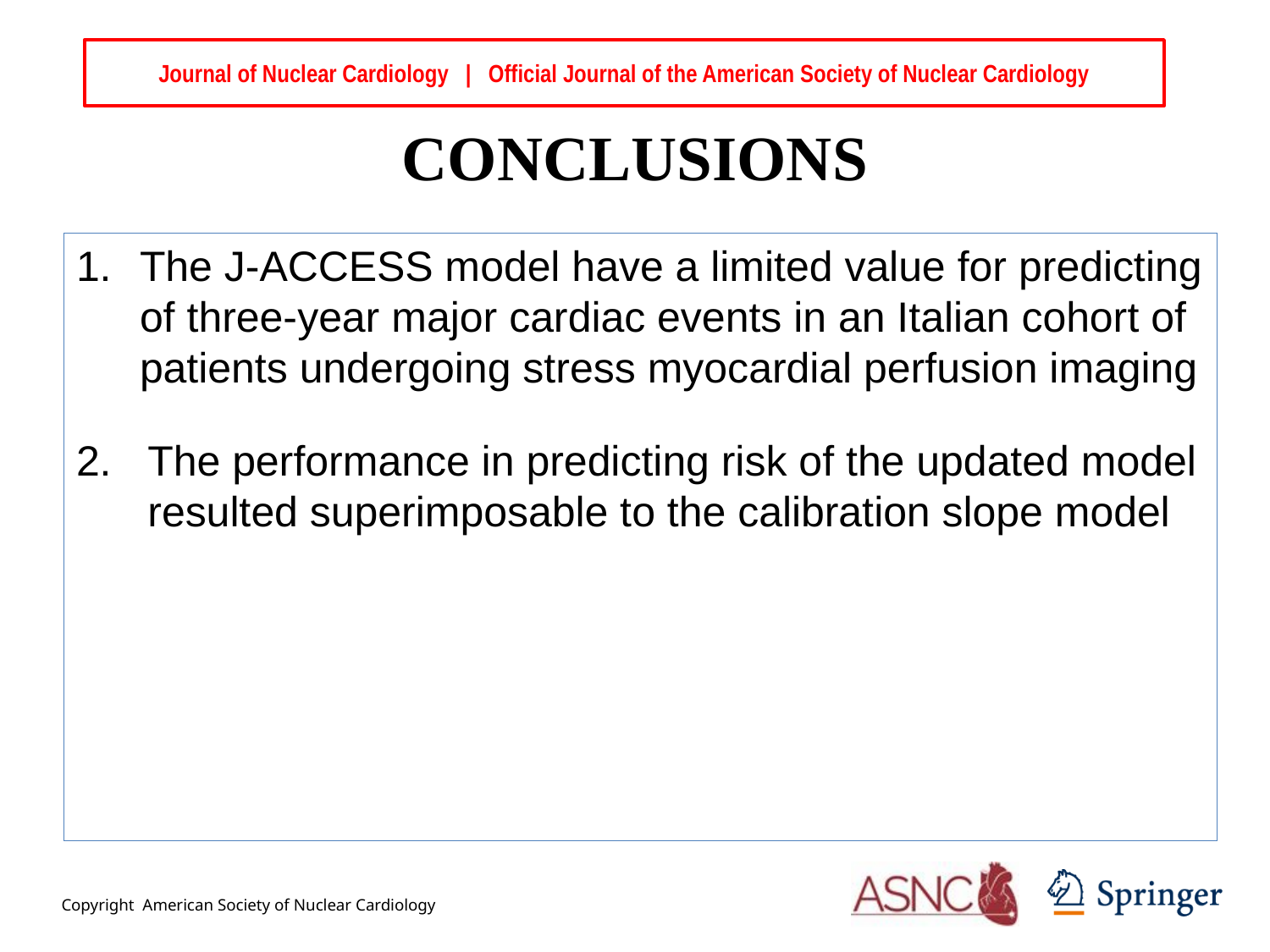

Journal of Nuclear Cardiology | Official Journal of the American Society of Nuclear Cardiology
# CONCLUSIONS
The J-ACCESS model have a limited value for predicting of three-year major cardiac events in an Italian cohort of patients undergoing stress myocardial perfusion imaging
The performance in predicting risk of the updated model resulted superimposable to the calibration slope model
Copyright American Society of Nuclear Cardiology
