# Supplementary material for: External validation and update of the J-ACCESS model in an Italian cohort of patients undergoing stress myocardial perfusion imaging
Source: J Nucl Cardiol. 2023 Jan 4;30(4):1443–53. doi: 10.1007/s12350-022-03173-4 (PMC10371932; doi:10.1007/s12350-022-03173-4)
Supplement: Supplementary file 3 — Supplementary file3 (PPTX 332 kb) [file 12350_2022_3173_MOESM3_ESM.pptx]

## Slide 1
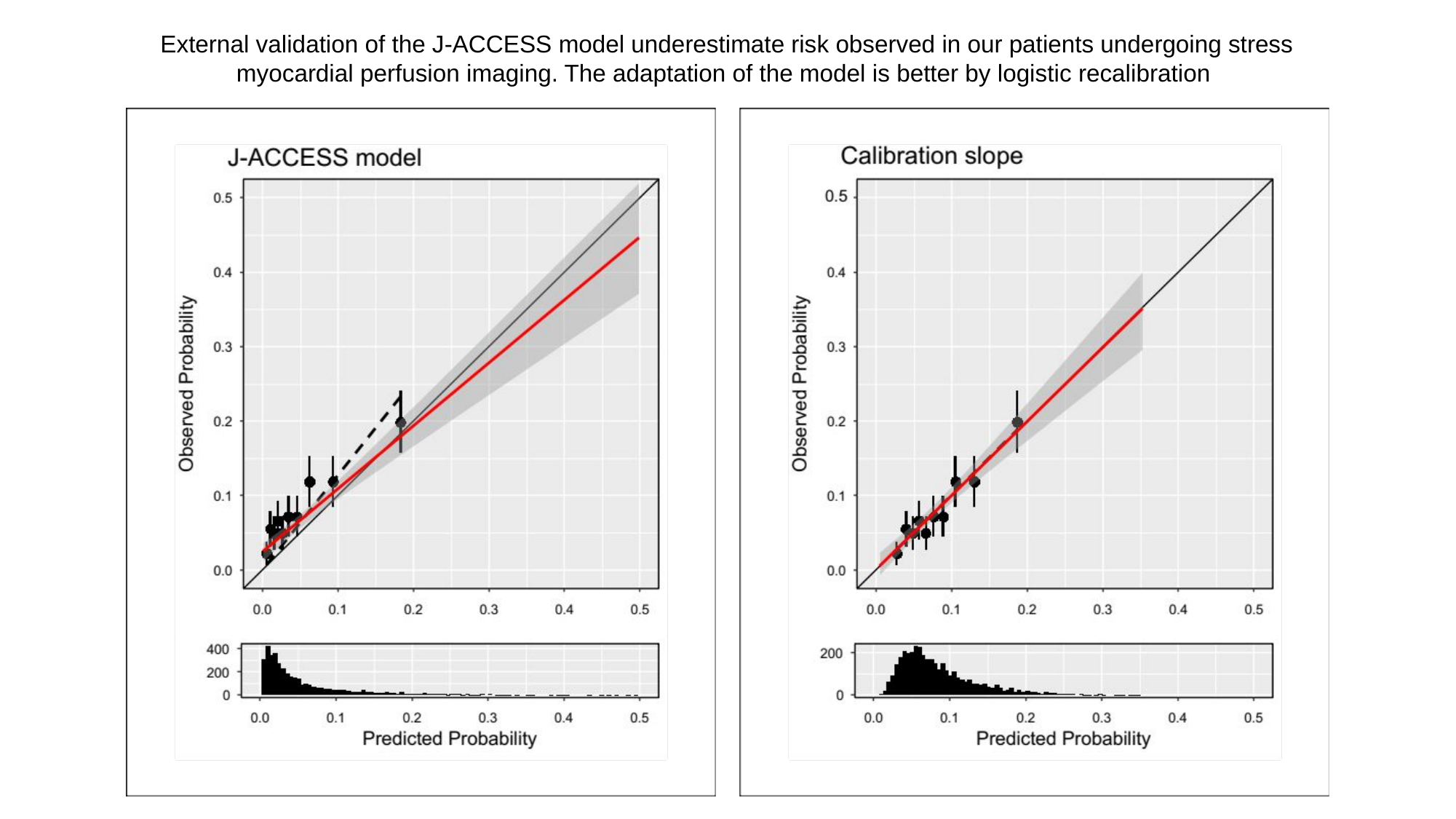

External validation of the J-ACCESS model underestimate risk observed in our patients undergoing stress myocardial perfusion imaging. The adaptation of the model is better by logistic recalibration
